# Impact of a double catastrophe, war and COVID-19, on health service utilization of a tertiary care hospital in Tigray: an interrupted time-series study

**DOI:** 10.1186/s13031-023-00537-6

**Published:** 2023-08-14

**Authors:** Hiluf Ebuy Abraha, Mengistu Hagazi Tequare, Hale Teka, Micheal Berhe Gebremedhin, Kibrom Gebreselassie Desta, Mohamedawel Mohamedniguss Ebrahim, Awol Yemane, Sintayehu Misgina Gebremariam, Kibrom Berhanu Gebresilassie, Tesfay Hailu Tekle, Mussie Tesfay Atsbaha, Ephrem Berhe, Bereket Berhe, Derbew Fikadu Berhe, Mulugeta Gebregziabher, L. Lewis Wall

**Affiliations:** 1 College of Health Sciences, Mekelle University, Tigray, Ethiopia; 2 Medical University of South Carolina, Charleston, SC USA; 3 Washington University in St. Louis, St. Louis, MO USA

**Keywords:** Armed conflict, COVID-19, Interrupted time-series analysis, Service utilization, Tigray, War

## Abstract

**Background:**

In developing nations with fragile healthcare systems, the effect of war is likely to be much worse than it would be in more developed countries. The presence of COVID-19 will also likely exacerbate the war’s impact. This study set out to determine the effect of armed conflict and the COVID-19 pandemic on health service utilization at Ayder Comprehensive Specialized Hospital, in the Tigray region of Ethiopia.

**Methods:**

An interrupted time-series study design was used to analyze patient visits over forty-eight consecutive months (from July 2017 to June 2021) at inpatient, outpatient, and emergency departments. Data were analyzed using segmented regression analysis with a defined outcome of level and trend changes in the number of patient visits. In addition, negative binomial regression analysis was also used to estimate the impact of both COVID-19 and the war on patient flow.

**Results:**

There were 59,935 admissions, 876,533 outpatient visits, and 127,872 emergency room visits. The effect of COVID-19 was seen as soon as the Tigray regional government imposed comprehensive restrictions. Immediately after COVID-19 appeared, all the service areas exhibited a significant monthly drop in visits; [-35.6% (95% CI: -48.2%, -23.1%)] for inpatient, [-60.6% (95% CI: -71.6%, -49.5%)] for outpatient, and [-44.1% (95% CI: -59.5%, -28.7%)] for emergency department visits. The impact of the war became apparent after a lag time of one month. Controlling the effects of time and COVID-19, the war led to a significant fall in inpatient visits [-44.3% (95% CI: -67.2%, -21.5%)], outpatients [-52.1% (95% CI: -82.7%, -21.5%)], and emergency-room attendances [-45.0% (95% CI: -74.8%, -15.2%)]. An upward trend in outpatient flow was observed after the war [1,219.4 (95% CI: 326.1, 2,112.8)].

**Conclusions:**

The present study has clearly indicated that the war and COVID-19 have led to a large reduction in admissions, outpatient attendance, and emergency department visits. The evidence from this study suggests that due to this double catastrophe, thousands of patients could not gain access to healthcare, with probable negative consequences. Governments and organizations should implement measures to buttress the healthcare system to maintain pre-war status of service.

**Supplementary Information:**

The online version contains supplementary material available at 10.1186/s13031-023-00537-6.

## Background

Global estimates show that over a billion people live in conflict-affected countries [1]. Armed conflict, which may well be the ultimate social determinant of health, has a profound impact on existing healthcare systems, in addition to its direct violent impact on civilian morbidity and mortality [2, 3]. It also puts both healthcare infrastructure and healthcare personnel at risk, due to security concerns. Additionally, armed conflict affects access to essential health services, impacts staffing levels, lowers the quality of care, reduces the availability of essential medicines and diagnostic services, leading to overall poorer health outcomes [4–7].

In developing nations, where the healthcare system is already weak and fragmented, the impact of war on the health sector is even more devastating [2, 8]. At the present time, the COVID-19 pandemic by itself has already severely affected healthcare systems globally. The pandemic has had a significant negative impact on maternal health, the utilization of family planning services, vaccination rates, and other essential health services. It has also caused a significant drop in overall service attendance for both diagnosis and treatment [9–13]. War worsens an already worsening situation.

War broke out in northern Ethiopia in November 2020. Assessment reports since then show that most healthcare facilities were destroyed and that the availability of basic medications including dialysis consumables and laboratory services dropped sharply. A sharp rise in food insecurity and the prevalence of malnutrition was also seen [14–17]. The war broke out while the COVID-19 pandemic was already challenging the ability of local healthcare system to function in the region [13, 18].

It is well-established that war often cripples the general ability of health systems to function, but knowing how it affects health service utilization during the COVID-19 pandemic is particularly important. Gesesew and colleagues have highlighted how war has impacted the health system in Tigray; however their study lacked data on healthcare utilization [16]. In the study area, no accessible published source to date has assessed the potential impact on health service utilization of the armed conflict during the unprecedented COVID-19 pandemic. We hypothesized that both calamities have caused a decrease in inpatient, outpatient, and emergency room visits. This study sought to estimate the effect of each of these factors on health service utilization at Ayder Comprehensive Specialist Hospital (ACSH) in the Tigray Region of northern Ethiopia.

## Methods

### Study area and period

This study was conducted at ACSH, a tertiary care hospital located in Mekelle, Tigray Region, Ethiopia. ACSH covers a large catchment area in northern Ethiopia [19]. The study period was between July 2017 and June 2021. July 2017 to March 2020 segmented into a pre-intervention period and April 2020 to June 2021 as a post-intervention period. The study period was set with reference to the Ethiopian fiscal year, which begins in July and ends the following June.

### Study design and population

The interrupted time-series design investigates all consecutive series of patients who visited the inpatient, outpatient, and emergency-room service areas. Four years of routinely collected health service data were considered. All records in these three service areas of the hospital were included in the analyses. A single patient may visit multiple service areas and may also have multiple follow-up visits at different times. Because the study focused on healthcare utilization, we used the number of patient visits as the primary input data.

### Outcome measures

The outcome measure is the number of visits to inpatient, outpatient, and emergency-room services. The outcome, measured in level and trend changes, estimates if there was a significant change in patient visits to the selected service areas because of the armed conflict and the COVID-19 pandemic compared to the pre-pandemic and pre-war states.

### Interventions

The interventions of interest were natural and man-made disasters. The first intervention was the COVID-19 pandemic and its restrictions. Following the first case detection in mid-March 2020 in Ethiopia, the regional government of Tigray declared a state of emergency on 26 March 2020, imposing travel restrictions and other containment measures [20]. In the analysis, the start of the first intervention, COVID-19 and its restrictions, was therefore considered April 1, 2020. The second intervention was the war, which broke out on November 4, 2020.

### Data collection and source

Data were acquired from the hospital health management information system (HMIS). Data were routinely collected monthly for the purpose of ordinary health management decision- making. Our data sources were the hospital patient registries.

### Data requirements and measurements

Various lines of evidence suggest different criteria regarding the number of data points required for an interrupted time-series study to be robust [21–23]. Our study has enough data points (forty-eight sequential months) to meet the necessary prerequisites for such a study. It is recommended that having long pre-intervention phase is beneficial, since it increases power to identify secular trends [24]. This study has thirty-three pre-intervention data points and has comparable time points for COVID-19 (seven) and war (eight) periods.

### Assumptions and potential biases

When conducting interrupted time series studies, it is important to check for autocorrelation and seasonality [23, 25]. Accordingly, there was no autocorrelation in the datasets, with a Durbin-Watson statistic value close to the center of its distribution (ranging between 1.8 and 2.1 for the three different models). Seasonality was examined using the least-squares regression model that includes a seasonal component; and there was no significant month to month seasonal pattern. In addition, visual inspection of the plotted data showed that there were no wild (astronomical) data points in the sequence of months.

### Data quality control

When these data were collected as a routine matter, a data quality control mechanism called lot quality assurance sampling (LQAS) was in place. LQAS is used to check the accuracy of HMIS data reporting. Data reported on HMIS forms and data recorded in registers should agree at least 85.0% of the time. This data quality control system was applied throughout both the pre- and post-intervention periods.

### Statistical analyses

Data were exported from the HMIS database of the hospital. An Excel spreadsheet was used for graphic presentations, and the rest were processed and analyzed using R statistical software [26]. First, descriptive statistics including measures of central tendency and dispersion were computed both for the baseline and intervention data. Trend status was represented using an annotated line graph. A counterfactual scenario i.e., the expected trend in the absence of both interventions, given the baseline trend, was predicted using the baseline trend’s regression model. Next, after organizing the dataset in an appropriate structure, a special method of interrupted time-series analysis known as segmented (piecewise) regression analysis [27] was employed to estimate the effect of war and COVID-19 on health services utilization. Though inpatient, outpatient, and emergency departments are interdependent with each other, we chose to compute three separate segmented regression analyses, as we want to estimate the effect of the disasters in each department.

In this segmented regression analysis, segments were divided into three portions. Using this analysis, we estimated the baseline level and trend of patient visits in each service area. In addition, the change in level and trend after occurrence of COVID-19 and the war was also examined. The following formula was used for the segmented regression with a couple of interventions.

$$\begin{gathered} Y(t) = {\beta _0} + {\beta _1}\left( {tim{e_{(t)}}} \right){\text{ }} + {\beta _2}\left( {COVID - {{19}_{(t)}}} \right){\text{ }} \hfill \\\,\,\,\,\,\,\,\,\,\,\,\,\,\,\,\,\, + {\beta _3}\left( {time{\text{ }}after{\text{ }}COVID - {{19}_{(t)}}} \right){\text{ }} + {\beta _4}\left( {wa{r_{(t)}}} \right){\text{ }} \hfill \\\,\,\,\,\,\,\,\,\,\,\,\,\,\,\,\,\, + {\beta _5}\left( {time{\text{ }}after{\text{ }}wa{r_{(t)}}} \right){\text{ }} + {e_t} \hfill \\ \end{gathered}$$

Y(t) represents number of visits per month at time t; $\beta$_*0*_ (baseline level) estimates number of patient visits per month at time zero; $\beta$_*1*_ (baseline trend) estimates change in the number of patient flow that occurs with each month before the intervention. *time*; time in months (1–48 months); $\beta$_*2*_ (level change due to COVID-19) estimates drop in the number of visits immediately after COVID-19, that is, from the end of the pre-intervention segment. $\beta$_*3*_ (trend change due to COVID-19) estimates the change in slope in the monthly number of patient visits after COVID-19, compared with the monthly trend before the pandemic.

$\beta$_*4*_ (level change due to war) estimates absolute change in the number of patient attendances immediately after war, from the end of the prior segment. $\beta$_*5*_ (trend change due to war) estimates the change in trend in the monthly number of patient flow after war, compared with the monthly trend before war. Finally, $e$_*t*_ (error term) represents the random variation which is not explained by the model. It is worth keeping in mind that, in this model, impact of war was estimated while controlling for the effect of COVID-19 with its restrictions; considering effect of COVID-19 continues the same way just like before the war broke out. Model fit for the regression models was checked for the three separate segmented regression analyses using adjusted R^2^ value. In addition, after checking the statistical dispersion of the count data, negative binomial regression was also conducted to estimate the effect of COVID-19 and the war on patient flow.

### Sensitivity analysis

Following the start of the war, all COVID-19 related restrictions were abandoned by default. In this scenario, sensitivity analysis was performed considering COVID-19 and its restrictions had no effect on patient visit during the war period. Using this secondary analysis, we expect the same finding with the first analysis, except for $\beta$_*4*_ and $\beta$_*5*_.

### Ethical considerations

The study protocol was reviewed and approved by the institutional review board of Mekelle University College of Health Sciences (#MU-IRB 1964/2022).

## Results

### Service utilization during the war and COVID-19

Over a million patient visits were included for analysis (59,935 admissions, 876,533 outpatient visits, and 127,872 emergency-room visits). The mean number of visits for the baseline periods of inpatient, outpatient, and emergency-room departments were 1,411 visits, 21,332 visits, and 2,912 visits per month respectively. After COVID-19 pandemic and the restrictive measures that followed, this figure dropped by 395(28.0%) in admission, 10,117(47.4%) in outpatient, and 634(21.8%) in emergency-room visits. The added effect of war brought pronounced reduction in admission 630(-44.6%), outpatient 9,575(-44.9%), and emergency-room 853(-29.3%) departments. Compared to the COVID-19 period, mean patient visit during war has also shown a decline in inpatient 235(-23.1%) and emergency-room 219(-9.6%) departments. However, 542(4.8%) increment in outpatient department visit was shown (Table 1).

**Table 1:** Patient visits during baseline period, war, and COVID-19 among inpatient, outpatient, and emergency departments, ACSH, Tigray, Ethiopia, 2017–2021 (N = 1,064,340)

Parameter	Intervention			Difference, n (%)		
	Pre	COVID-19	War	COVID-19 vs. Pre	War vs. Pre	War vs. COVID-19
**Inpatient**						
Total number of visits	46,578	7,109	6,248			
Mean [SD]	1,411[101]	1,016[138]	781[201]	-395(-28.0)	-630(-44.6)	-235(-23.1)
Range	1,256-1,703	782-1,147	346-1,011			
**Outpatient**						
Total number of visits	703,971	78,505	94,057			
Mean [SD]	21,332[2,860]	11,215[2,457]	11,757[4,355]	-10,117(-47.4)	-9,575 (-44.9)	+ 542(+ 4.8)
Range	16,258 − 28,523	7,973 − 13,943	4,228 − 16,297			
**Emergency**						
Total number of visits	96,144	15,254	16,474			
Mean [SD]	2,912[447]	2,278[459]	2,059[655]	-634(-21.8)	-853(-29.3)	-219(-9.6)
Range	1,966-3,854	1,522-2,967	825-2,924			

SD: standard deviation, COVID-19 vs. Pre: mean difference during COVID-19 compared to pre-intervention period, War vs. Pre: mean difference during war compared to pre-intervention period, War vs. COVID-19: mean difference during war compared to the COVID-19 period

### Effect of COVID-19

**Fig. 1:**
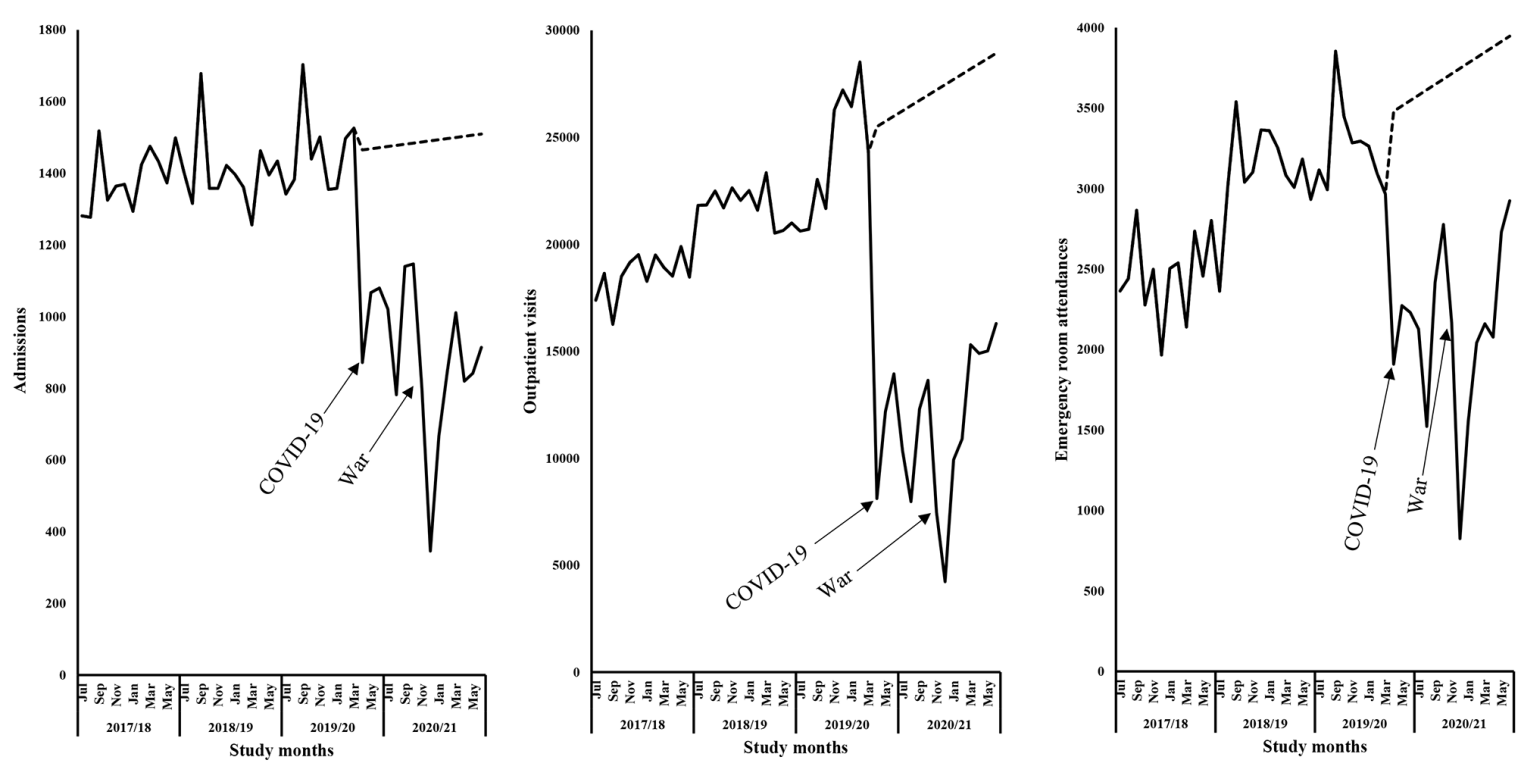
Trend of admissions, outpatient departments, and emergency room attendances, Ayder comprehensive specialized hospital, Tigray, Ethiopia, 2017–2021. Bold line represents actual admissions, outpatient and emergency visits and dashed line represents forecasted counterfactual state

In all the service areas, the effect of COVID-19 was seen immediately after the start of the outbreak and the restrictions that were imposed. Immediately after COVID-19, sharp declines in inpatient, outpatient, and emergency-room patient visits were observed (Fig. 1).

In the inpatient department, before the beginning of the observation period there were 1,361 patient visits per month on average, with a 95% confidence interval (CI) of (1,279.5, 1,442.2). Right after COVID-19, the number of inpatient admissions showed a significant immediate drop [-521.7(95% CI: -705.5, -337.9)]. Outpatient visits were showing positive level [17,408.9(95% CI: 16,162.5, 18,655.3)] and trend [245.2(95% CI: 178.3, 312.2)] changes during the baseline period. In the outpatient department, COVID-19 and the restrictions imposed caused a drastic reduction visit [-15,448.5(95% CI: -18,263.9, -12,633.1)] without significant trend change. In the emergency department, before the beginning of the series there was an average 2,380 monthly patient visits (95% CI: 2,142.6, 2,617.6). Unlike the inpatient service, the emergency-room showed a sharp increase in month-to-month trend in patient flow during baseline period [33.3(95% CI: 20.6, 46.1)]. Following COVID-19, a significant immediate absolute reduction in emergency-room attendance was observed [-1,534.7(95% CI: -2,071.3, -998.2)] (Table 2).

Table 3 provides the summary statistics for percent level change in health services utilization. Following COVID-19, 35.6% immediate reduction in admission status was observed (95% CI: -48.2%, -23.1%). As a result of COVID-19, the number of outpatient visits decreased by more than half [-60.6% (95% CI: -71.6%, -49.5%)]. COVID-19 also led to a significant immediate drop in emergency-room attendances [-44.1% (95% CI: -59.5%, -28.7%)].

**Table 2:** Segmented regression analysis assessing the impact of war and COVID-19 on patient visits at inpatient, outpatient, and emergency departments, ACSH, Tigray, Ethiopia, 2017–2021 (N = 1,064,340)

Service area	Coefficient (95% CI)	SE	t-test	P-value	R^2^-value
**Inpatient**					
Baseline level$, \beta$_*0*_	1,360.9(1,279.5, 1,442.2)	40.3	33.7	< 0.001	0.84
Baseline trend,$\beta$_1_	3.2(-1.2, 7.5)	2.2	1.5	0.152	
Level change after COVID-19, $\beta$_*2*_	-521.7(-705.5, -337.9)	91.1	-5.7	< 0.001	
Trend change after COVID-19,$\beta$_*3*_	20.9(-24.5, 66.3)	22.5	0.9	0.359	
Level change after war, $\beta$_*4*_	-493.0(-747.3, -238.8)	126.0	-3.9	< 0.001	
Trend change after war,$\beta$_*5*_	22.3(-36.0, 80.7)	28.9	0.8	0.444	
**Outpatient**					
Baseline level$, \beta$_*0*_	17,408.9(16,162.5, 18,655.3)	617.6	28.2	< 0.001	0.90
Baseline trend,$\beta$_1_	245.2(178.3, 312.2)	33.2	7.4	< 0.001	
Level change after COVID-19, $\beta$_*2*_	-15,448.5(-18,263.9, -12,633.1)	1,395.1	-11.1	< 0.001	
Trend change after COVID-19,$\beta$_*3*_	142.2(-553.0, 837.4)	344.5	0.4	0.682	
Level change after war, $\beta$_*4*_	-6,631.6 (-10,525.7, -2,737.5)	1,929.6	-3.4	0.001	
Trend change after war,$\beta$_*5*_	1,219.4(326.1, 2,112.8)	442.7	2.7	0.009	
**Emergency**					
Baseline level$, \beta$_*0*_	2,380.1(2,142.6, 2,617.6)	117.7	20.2	< 0.001	0.70
Baseline trend,$\beta$_1_	33.3(20.6, 46.1)	6.3	5.3	< 0.001	
Level change after COVID-19, $\beta$_*2*_	-1,534.7(-2,071.3, -998.2)	265.8.0	-5.7	< 0.001	
Trend change after COVID-19,$\beta$_*3*_	44.6(-87.9, 177.1)	65.6	0.7	0.501	
Level change after war, $\beta$_*4*_	-1,120.4(-1,862.5, -378.4)	367.7	-3.0	0.004	
Trend change after war,$\beta$_*5*_	118.8(-51.4, 289.1)	84.4	1.4	0.166	

CI: Confidence interval, SE: Standard error

### Effect of the war

Unlike that of COVID-19, the impact of war was not seen immediately, but after a time-lag. Inpatient, outpatient, and emergency-room departments all showed an obvious decrease after the war broke out (Fig. 1). Adjusting for the effect of time and COVID-19, the war created a large reduction in health service utilization in all service areas. Right after the war, inpatient flow significantly dropped by 493 patients per month (95% CI: -747.3, -238.8). In addition to the effect of COVID-19, patient visits in the outpatient department were severely affected by the war, with large immediate absolute reduction in the total visits [-6,631.6(95% CI: -10,525.7, -2,737.5)]. Following the war, a positive trend change in outpatient visits was also observed [1,219.4(95% CI: 326.1, 2,112.8)]. As is shown in Table 2, the start of the war led to a drop of 1,120 in patient visits in the emergency-room (95% CI: -1,862.5, -378.4). After the start of the war, however, there was no statistically significant trend change in emergency-room visits.

Percent change estimation shows the war led to 44.3% immediate reduction in admission status (95% CI: -67.2%, -21.5%). The war has also brought a large reduction in the number of outpatient visits [-52.1% (95% CI: -82.7%, -21.5%)]. Likewise, nearly half (45.0%) war caused immediate reduction in emergency-room attendance was also seen (95% CI: -74.8%, -15.2%) (Table 3). The predicted counterfactual scenario (for both COVID-19 and war) of the outpatient and emergency visits shows a pronounced rise in slope, while admission status exhibited a more stationary slope (Fig. 1).

**Table 3:** Percent change in patient visit of inpatient, outpatient, and emergency departments associated with the war and COVID-19, ACSH, Tigray, Ethiopia, 2017–2021 (N = 1,064,340)

Service area	Coefficient (95% CI)	P-value
**Inpatient**		
Level change after COVID-19, $\beta$_*2*_	-35.6% (-48.2%, -23.1%)	< 0.001
Level change after war, $\beta$_*4*_	-44.3% (-67.2%, -21.5%)	< 0.001
**Outpatient**		
Level change after COVID-19, $\beta$_*2*_	-60.6% (-71.6%, -49.5%)	< 0.001
Level change after war, $\beta$_*4*_	-52.1% (-82.7%, -21.5%)	0.001
**Emergency**		
Level change after COVID-19, $\beta$_*2*_	-44.1% (-59.5%, -28.7%)	< 0.001
Level change after war, $\beta$_*4*_	-45.0% (-74.8%, -15.2%)	0.004

CI: Confidence interval, SE: Standard error

### Sensitivity analysis findings

What affects service utilization is not just COVID-19 itself, but mostly the containment measures imposed, including travel restrictions and lockdowns. We carried out a secondary analysis as COVID-19 restrictions were abandoned after the start of war. War brought a significant level change compared to our first analysis, where the impact of COVID-19 was presumed to continue similarly after the war broke out. Accordingly, after the war began the number of admissions dropped by 869 patients per month. The war has also led to an estimated immediate absolute reduction of 21,085 and 2,343 patient visits for outpatient and emergency-room departments respectively. A significant jump in trend was also seen in all the service areas, following the war (Table 4). In the negative binomial regression analysis, following COVID-19, inpatient patient flow dropped by 33.0% [Incidence Rate Ratio (IRR) = 0.67; 95% CI:0.58,0.78], and outpatient and ER flow also decreased by 60.0% [IRR = 0.40; 95% CI:0.33,0.48] and 42.0% [IRR = 0.58; 95% CI:0.48,0.69] respectively. War brought 49.0% decrement in admissions, 62.0% in outpatient flow, and 51.0% in ER attendance (**Supplement** Table 1).

**Table 4:** Sensitivity analysis on the impact of the war on patient visits in the inpatient, outpatient, and emergency departments, ACSH, Tigray, Ethiopia, 2017–2021 (N = 1,064,340)

Service area	Coefficient (95% CI)	SE	t-test	P-value
**Inpatient**				
Level change after war, $\beta$_*4*_	-868.7(-1,059.8, -677.5.0)	94.7	-9.2	< 0.001
Trend change due to war, $\beta$_*5*_	43.2(6.1, 80.4)	18.4	2.3	0.024
**Outpatient**				
Level change after war, $\beta$_*4*_	-21,084.7(-24,012.9, -18,156.5)	1,451.0	-14.5	< 0.001
Trend change due to war, $\beta$_*5*_	1,361.6(792.7, 1930.6)	281.9	4.8	< 0.001
**Emergency**				
Level change after war, $\beta$_*4*_	-2,343.0(-2,901.0, -1,785.0)	276.5	-8.4	< 0.001
Trend change due to war, $\beta$_*5*_	163.5(55.0, 271.9)	53.7	3.0	0.004

CI: Confidence interval, SE: Standard error

## Discussion

This interrupted time-series study investigates if the double catastrophes of war and COVID-19 influenced health services utilization at a tertiary care hospital in northern Ethiopia. Our study demonstrates that both COVID-19 with the restrictions that it imposed, and the recent war have led to significant absolute reduction in inpatient, outpatient, and emergency-room visits.

Previous reports from both developed and developing nations have demonstrated that COVID-19 has had a negative impact on health service utilization, which led to a significant drop in admission, outpatient and emergency attendances [9, 11, 12, 28, 29]. This is consistent with our findings that COVID-19 caused a significant absolute decrease in all the three service areas. In a pre-post study from Tigray, in agreement with our findings, the number of admissions decreased during COVID-19 pandemic; however, emergency unit attendance showed increase in patient visit, contrary to the current study [13]. This rather contradictory result can be explained in part by the difference in the type of patients they serve and study facilities the studies were undertaken, given our study hospital was a referral specialized hospital, unlike the comparative study. The difference in length of time the studies took into account might also explain the discrepancy. In this study, the large reductions in patient visits due to COVID-19 may be related to the government’s low capacity for prevention and surveillance actions, as well as misinformation at the population level [30]. In Tigray, COVID-19 patients were being treated in a dedicated COVID-19 isolation and treatment centers [31, 32]. It is, therefore, likely that this might also have affected the patient flow negatively as the study hospital was not receiving COVID-19 patients.

Our finding also suggests that the utilization of health services which were severely stricken by COVID-19 was then affected further by the war. According to both the first and second (sensitivity) analyses, the armed conflict in the region caused a significant drop in patient visits in all the three service areas. A prior report from Syria showed an inverse relationship between conflict-related incidents and routine health service utilization, echoing our findings [33]. Studies have shown that people who live in a war zones face significant challenges accessing healthcare services due to transportation problems and the costs of medical care [34, 35]. The extreme decline in admissions, and the decrease in utilization of both outpatient and emergency-room services may be attributable to markedly deceased access to healthcare due to transportation problems and the disruption of referrals caused by the war. In addition, financial costs are another challenge for our population, as community-based health insurance schemes are no longer functioning as a result of the war [16]. One of the main reasons for emergency attendances drop during wartime could likely be due to fear of attacks. A recent study showed that there was a significant drop in institutional delivery during the war time. Mothers were giving birth not only at home but also at bushes, trying to hide from the attacks [36].

In the baseline data from all the departments, data for September is slightly higher compared to other months. This was due to the Ethiopian calendar system, which has a 13th month called Pagume, with only 5 days and 6 days in a leap year. These 5 or 6 leap-year calendar days come just before September [37]. In the Ethiopian reporting system, data from the month of Pagume are added to September.

Unlike the effect of COVID-19, which was almost immediately, the impact of the war was not observed until a month after hostilities began. The delayed impact is presumably due to the reason that the armed conflict was escalating initially until it affected the entire region after the first month of conflict had passed (December), in which the fewest number of visits were observed. In addition, there was mass flow of war casualties to the emergency-room in the first month of the war, which explains why service utilization in that month (November 2020) wasn’t much affected.

After the start of the war, there was a significant drop in outpatient visitations, but the trend increased positively thereafter. Similar increasing trend was seen in admission status and emergency-room attendances too, although the inclination was not statistically significant. This gradual rise in patient visits may be due to the surge in the number of internally displaced people in the study area. In addition, when the war began intensifying and widening, it led to the partial or complete non-functioning of most health facilities in the region, disabling or destroying 70% of the hospitals and 85% of the health centers in Tigray [16]. Given that the study hospital, ACSH, was among the few hospitals still functioning, patients had no choice but to reach such a facility, even if they had financial or transport problems.

During COVID-19 period, though patient visits dropped significantly, patients still had a chance to visit their nearby health facilities. During the time of war, on the other hand, patients were unable to access even primary healthcare services, given vast majority of health facilities were not functioning [16]. Overall, this study supports the impression that the ongoing armed conflict added to the combination of COVID-19 with its restrictions, reduced healthcare access for thousands of patients who could not receive inpatient, outpatient, or emergency-room services.

Currently, WHO has announced that COVID-19 is no longer a public health emergency [38] and an agreement has been made between the warring parties in Ethiopia [39]. While evidence has shown an increase in COVID-19 vaccine uptake in Tigray region [40], the war brought problems in the region is still not fully resolved. More than seven months into the agreement, a recent reports showed that displaced people did not still get a chance to go back home and people are dying of hunger in need of food and healthcare access [41, 42].

### Strength and limitation of the study

The strengths of the current study include the use of a time-series design, which is a powerful quasi-experimental study design to investigate the effect of an intervention where randomization is not possible [25]. The time-series nature of our dataset made it possible to know the exact month when the impact of the interventions was seen. As in our case, the use of data routinely collected for other purposes is both fortuitous as well as appropriate for time-series studies [23]. The use of a routinely collected dataset from a very large hospital (over a million observations) is a further strength of our study. The lack of a control group, and not having other variables to control such as health seeking behavior of the patients is a limitation of the study.

## Conclusions

In summary, this study has shown that both the war and the COVID-19 pandemic have had negative effects on the utilization of healthcare services at ACSH. This double catastrophe has led to large reductions in inpatient admissions to the hospital, in outpatient utilization, as well as visits to the emergency room. The impact of the war was apparent even considering COVID-19 still had an effect after the war broke out. It is imperative that efforts be made by local, regional, and national government bodies, as well as by international aid organizations to maintain the pre-war state of the healthcare system in Tigray. The civilian people are innocent bystanders to the armed conflict. They must receive better healthcare services and have their rights respected under these difficult circumstances.

### Electronic supplementary material

Below is the link to the electronic supplementary material.

Supplementary Material 1

## Data Availability

The dataset is available from the corresponding author on reasonable request.
