# Supplementary material for: Impact of a double catastrophe, war and COVID-19, on health service utilization of a tertiary care hospital in Tigray: an interrupted time-series study
Source: Confl Health. 2023 Aug 14;17:37. doi: 10.1186/s13031-023-00537-6 (PMC10426210; doi:10.1186/s13031-023-00537-6)
Supplement: Supplementary file 1 — Supplementary Material 1 [file 13031_2023_537_MOESM1_ESM.docx]

| Variables Category | | IRR (95% CI) | P-Value |
| --- | --- | --- | --- |
| Inpatient | | | |
|  | Time | 1.00(1.00,1.01) | 0.180 |
|  | COVID-19 | 0.67(0.58,0.78) | <0.001 |
|  | WAR | 0.51(0.43,0.60) | <0.001 |
| OPD | | | |
|  | Time | 1.01(1.01,1.02) | <0.001 |
|  | COVID-19 | 0.40(0.33,0.49) | <0.001 |
|  | WAR | 0.38(0.30,0.48) | <0.001 |
| ER | | | |
|  | Time | 1.01(1.01,1.02) | <0.001 |
|  | COVID-19 | 0.58(0.48,0.69) | <0.001 |
|  | WAR | 0.49(0.40,0.61) | <0.001 |

Supplement Table 1: Negative binomial regression analysis assessing the impact of war and COVID-19 on patient visits at inpatient, outpatient, and emergency departments, ACSH, Tigray, Ethiopia, 2017-2021 (N=1,064,340).

ER: Emergency room, IRR: Incidence Rate Ratio, ER: Emergency Room. After finding out that our dataset was extremely over dispersed (with the minimum residual deviance ratio degree of freedom greater than 16) we run negative binomial regression analysis as per the rule. The analysis was run with the consideration of zero COVID-19 restrictions impact after the war broke out.
